# A multimodal EEG-based psychological risk representation method using spatiotemporal graph attention and adaptive gated fusion

**DOI:** 10.3389/fpsyt.2026.1886039

**Published:** 2026-08-28

**Authors:** Yufang Wu, Guangpeng Zhang, Guofeng Yu

**Affiliations:** 1 Quzhou College of Technology, Quzhou, China; 2 Department of Neurosurgery, Quzhou People's Hospital; The Quzhou Affiliated Hospital, Wenzhou Medical University, Quzhou, China

**Keywords:** cross-modal fusion, EEG, explainability, mental health assessment, risk representation, spatiotemporal graph attention network

## Abstract

**Objective:**

As mental health issues gain increasing attention, traditional risk assessment methods based on psychological questionnaires are limited by subjective biases and a narrow dimensionality of information, making them difficult to meet the needs of precision medicine. To address this challenge, this study proposes a cross-modal temporal representation method that integrates spatiotemporal graph attention with an adaptive gating mechanism.

**Methods:**

The method aims to dynamically correct and provide explainable analysis of the health risk labels generated from psychological profiling using objective electroencephalogram (EEG) signals. Specifically, the study first constructs individual psychological profiles based on the raw Big Five personality scores from the AMIGOS public dataset and generates initial health risk labels in combination with self-reported emotional data. Subsequently, for the 14-channel EEG signals, a cascaded architecture combining graph attention networks and bidirectional long short-term memory networks is designed to effectively extract spatial topological features and temporal dynamic information from brain regions. Finally, a cross-modal attention mechanism and a temporal gating fusion module are introduced to deeply integrate static psychological features with dynamic neural signals, thereby refining the representation of risk labels.

**Results:**

Experimental results demonstrate that the proposed method significantly improves the accuracy and robustness of risk prediction on the AMIGOS dataset, effectively overcoming the subjective interference of questionnaire-based assessments.

**Conclusions:**

Additionally, through quantitative analysis of attention weights, this study reveals the contribution patterns of key EEG features to risk prediction, providing an objective basis for personalized psychological intervention strategies and offering a new technical pathway for low-cost, interpretable multimodal mental health assessment.

## 1 Introduction

With the advancement of precision and objective mental health assessment, the integration of multimodal physiological and psychological information has become an emerging research direction in psychological science, neuroengineering, and intelligent computing (1–3). Among existing approaches, psychological profiling based on the Big Five personality model has been widely applied in mental health risk assessment due to its effectiveness in characterizing stable individual psychological traits (4, 5). These assessments provide important references for risk modeling and personalized intervention strategies. However, conventional profiling methods primarily rely on self-reported questionnaires, which are inevitably affected by subjective cognitive bias, response concealment, and limited information dimensions. Consequently, the generated risk assessments may not fully reflect underlying neurophysiological states and individual psychological variations (6, 7). Therefore, integrating psychological profiling with objective physiological signals to improve the reliability and interpretability of risk representation has become an important challenge in intelligent mental health assessment. With recent advances in EEG acquisition technologies and deep learning algorithms, cross-modal fusion approaches combining psychological information and neural signals provide a promising solution for overcoming the limitations of traditional assessments. By leveraging complementary information from different modalities, such methods offer new opportunities for adaptive risk representation and explainable mental health analysis.

Currently, psychological profiling techniques based on the Big Five personality model have established a relatively mature application framework in mental health assessment. Existing studies mainly improve the consistency and generalizability of risk-related labels by optimizing the reliability and validity of assessment scales and constructing association models between personality traits and psychological risks. These approaches have been widely applied to characterize stable psychological traits and support risk prediction. However, such methods fundamentally rely on subjective information obtained from questionnaires or self-reported scales, which limits their ability to capture transient psychological state variations and underlying physiological mechanisms.

For example, Xu et al. (8) developed a scale-based risk assessment framework for elderly internet healthcare services, achieving differentiated risk management through the analysis of relationships between personality traits and risk exposure. Norman et al. (9) investigated the psychometric properties of a moral injury scale in high-risk occupational populations and improved the scoring mechanism by incorporating Big Five personality dimensions. Cai et al. (10) explored automatic personality recognition based on facial video action features, demonstrating the feasibility of personality inference without questionnaire-based assessments. Although these studies have advanced psychological profiling modeling, they mainly depend on behavioral representations or explicit feature inputs, making it difficult to reveal the underlying neurophysiological mechanisms. Compared with psychological assessments, electroencephalography (EEG), as an objective physiological signal reflecting neural activity, provides millisecond-level temporal resolution and real-time acquisition capability, and has been widely applied in emotion recognition, risk assessment, and neural state analysis. However, EEG signals suffer from strong noise interference, low signal-to-noise ratios, and substantial inter-individual variability, which remain major challenges for stable personality modeling and cross-condition generalization. Sharma et al. (11) combined graph signal processing with deep learning techniques and improved EEG analysis performance through adaptive denoising and topological feature enhancement. Cai et al. (12) proposed an EEG representation method based on azimuthal equidistant projection and employed Swin Transformer to model spatiotemporal features. Hameed et al. (13) investigated the integration of EEG and brain–computer interface techniques in motor imagery tasks, demonstrating the potential of EEG for neural cognitive decoding. Despite continuous advances in EEG feature representation, these approaches still suffer from a considerable semantic gap between neural signal patterns and high-level psychological profiling. With the development of multimodal fusion technologies, researchers have begun to integrate psychological questionnaires with EEG signals to construct joint modeling frameworks, aiming to improve the objectivity and robustness of personality assessment. Guleva (14) developed an EEGNet-based personality classification model and verified the feasibility of deep learning for EEG-based personality recognition. Jach et al. (15) decoded Big Five personality scores using resting-state EEG power spectra and revealed associations between EEG frequency characteristics and personality traits. Li et al. (16) employed regression models to predict personality scores and validated their effectiveness through cross-validation. Riaz et al. (17) constructed classification models based on EEG data collected during stress tasks, further supporting the relationship between personality traits and neural oscillatory patterns. These studies have demonstrated the feasibility of jointly modeling psychological traits and neural signals to some extent. However, most existing multimodal approaches still rely on feature concatenation or static fusion strategies (18), lacking deep alignment mechanisms between personality traits and dynamic EEG spatiotemporal patterns. Consequently, they are limited in capturing the complex interactive relationships between psychological characteristics and neural activities. Moreover, under conditions involving noise interference and individual variability, existing fusion strategies often exhibit insufficient adaptive capability, restricting their practical application in real-world mental health assessment scenarios. Therefore, developing dynamic collaborative modeling approaches that effectively bridge psychological profiling and EEG signals while fully exploiting the intrinsic correlations between heterogeneous modalities has become a critical issue for advancing objective and precise mental health assessment.

To address these challenges, this study proposes a cross-modal temporal prediction framework based on the AMIGOS public dataset (19), integrating spatiotemporal graph attention mechanisms (20) (GAT) and adaptive gating strategies (21) to optimize health risk label generation based on psychological profiling. Specifically, a preprocessing pipeline including baseline correction, resampling, and band-pass filtering is first applied to 14-channel EEG signals. A graph attention network (GAT) is then employed to model brain-region topological relationships and extract spatial dependency features. Subsequently, a bidirectional long short-term memory network (Bi-LSTM) (22) is introduced to capture temporal dynamics, enabling joint representation learning of EEG spatiotemporal features. Based on this representation, a personality-guided cross-modal attention mechanism is designed, where Big Five personality features are used as queries to guide the selective alignment of EEG spatiotemporal representations, facilitating deep interaction between personality semantics and neural activity patterns (23). Finally, an adaptive gated fusion module is developed to dynamically regulate the contributions of different modalities under noisy conditions, improving model robustness. Meanwhile, attention weights are utilized to provide interpretability by identifying critical brain regions and informative temporal segments.

The main contributions of this study are summarized as follows:

1. We propose a cascaded EEG feature extraction framework that captures both spatial topology of brain regions and temporal dynamics, fully representing the spatiotemporal coordination of EEG signals and overcoming the dimensional limitations of traditional feature extraction methods.
2. By integrating static psychological profiles with dynamic EEG signals in a deep, synergistic manner, the method addresses the limitations of shallow fusion and poor dynamic adaptability in traditional approaches, improving both accuracy and robustness of risk label representation.
3. Quantitative analysis of attention weights reveals the neural indicators associated with different personality traits, filling the gap in explainability of existing cross-modal models and providing an objective basis for personalized intervention strategies.

## 2 Theoretical and technical foundations

### 2.1 Graph attention network

The multi-channel acquisition nature of EEG signals endows them with an inherent graph structure, where each electrode corresponds to a node in the graph, and the physical distances and functional connections between electrodes define the edges. Graph Attention Network (GAT) models the dependencies among nodes through an adaptive attention mechanism, overcoming the limitations of traditional approaches in capturing the spatial topology of brain regions and accurately extracting collaborative features across EEG channels (24).

In the graph-structured modeling, we define the EEG graph structure as *G* = (*V*, *E*, *A*), where $V=\left\{v_{1},v_{2},\ldots ,v_{N}\right\}$ represents the set of nodes corresponding to the 14 EEG channels; *v_i_* denotes the feature vector *x_i_* of the *i*-th channel; *E* is the set of edges representing the connectivity between channels; and *A* is the adjacency matrix, constructed based on the physical distances between electrodes using a Gaussian kernel function. The specific mathematical formulation is as follows (Equation 1):

(1) $$A_{ij}=\exp\left(-\frac{d_{ij}^{2}}{2\sigma^{2}}\right)\cdot ∥(d_{ij}\leq d_{0})$$

Where *d_ij_* represents the scalp distance between channels *i* and *j*; σ is the bandwidth of the Gaussian kernel; *d*_0_ denotes the maximum connection threshold; and ||(.) is the indicator function.

In the attention mechanism, during the process of feature enhancement and transformation, a shared weight matrix *W* is used to linearly transform the node features, while a bias term *b* is introduced to improve the expressive capacity (Equation 2):

(2) $$h_{i}=\text{ReLU}(Wx_{i}+b)$$

Where *h_i_* denotes the transformed features, and ReLU represents the activation function.

In the calculation of attention coefficients, a parameterized attention scoring function is employed, where a weight vector *a* is used to capture the feature correlations between nodes and (*i*,*j*). Layer normalization is incorporated to alleviate gradient vanishing (Equation 3).

(3) $$e_{ij}=a^{T}\cdot \text{LN}\left(\left[h_{i}∥h_{j}\right]\right)$$

Where *a* denotes the attention weight vector, || represents vector concatenation, LN(.) denotes the layer normalization operation and *e_ij_* is the attention coefficient.

To enhance feature robustness, multi-head attention with K parallel heads is employed, where each head independently learns attention weights. The resulting features are fused through concatenation, and L2 regularization is applied to constrain the parameters (Equation 4).

(4) $$h_{i}^{\prime }=\prod_{k=1}^{K}\sigma \left(\sum_{j}\alpha_{ij}^{k}h_{j}^{k}\right)+\lambda {\left\|W\right\|}_{2}^{2}$$

Where $\alpha_{ij}^{k},h_{j}^{k}$ denote the attention weights and transformed features of the *k*-th head, respectively; σ is the Sigmoid activation function, and λ represents the regularization coefficient.

### 2.2 Bidirectional long short term memory network

The spatial topological features extracted by GAT still constitute a temporal sequence, and their dynamic fluctuations, reflecting changes in brain region coordination across different time windows, cannot be captured through spatial modeling alone. Therefore, in this study, a bidirectional long short-term memory network (Bi-LSTM) is employed to address the gradient vanishing problem inherent in traditional RNNs (25) through its gating mechanism, while simultaneously integrating forward and backward temporal information to achieve a comprehensive representation of EEG spatiotemporal features.

In the core gating computations of a unidirectional LSTM, at a single time step t, the LSTM takes the GAT-extracted feature *z_t_* as input, and updates its states by combining the previous hidden state *s_t_*_−1_ and cell state *c_t_*_−1_. The computations for each module are as follows (Equation 5):

(5) $$\begin{array}{l} g_{t}=\sigma \left(W_{g}\cdot [s_{t-1};z_{t}]+b_{g}\right)\\ i_{t}=\sigma \left(W_{i}\cdot [s_{t-1};z_{t}]+b_{i}\right)\\ {\hat{c}}_{t}=\text{Tanh}\left(W_{c}\cdot [s_{t-1};z_{t}]+b_{c}\right)\\ c_{t}=g_{t}\circ c_{t-1}+i_{t}\circ {\hat{c}}_{t}\\ o_{t}=\sigma \left(W_{o}\cdot [s_{t-1};z_{t}]+b_{o}\right)\\ s_{t}=o_{t}\circ \text{Tanh}(c_{t}) \end{array}$$

Where g*_t_* denotes the forget gate weight vector; **i***_t_* represents the input weight for the current spatial feature; ${\hat{c}}_{t}$ is the candidate cell state; σ is the activation function; $W_{g},W_{i},W_{c}$ and $b_{g},b_{i},b_{c}$ are the corresponding weight matrix and bias term, respectively; ∘ denotes element-wise multiplication; **o***_t_* regulates the cell state output; and **s***_t_* represents the unidirectional temporal feature.

For the Bi-LSTM, forward and backward branches are constructed in parallel to perform bidirectional modeling of the GAT-extracted sequence (Equation 6):

(6) $$\begin{array}{l} {\vec{s}}_{t}=\vec{LSTM}\left(z_{t},{\vec{s}}_{t-1}\right)\;(t=1,2,\ldots ,T)\\ {\overset{\leftarrow }{s}}_{t}=\overset{\leftarrow }{LSTM}\left(z_{t},{\overset{\leftarrow }{s}}_{t+1}\right)\;(t=T,T-1,\ldots ,1)\\ s_{t}=\left[{\vec{s}}_{t};{\overset{\leftarrow }{s}}_{t}\right] \end{array}$$

Where ${\vec{s}}_{t}$ captures the evolution trajectory of past-to-current spatial features, ${\overset{\leftarrow }{s}}_{t}$ captures the future-to-current temporal feedback correlations, and s*_t_* represents the concatenated features integrating information from both directions.

### 2.3 Cross modal attention and gating fusion

The EEG spatiotemporal fusion features **s***_t_* extracted by the GAT–BiLSTM and the MLP-encoded static features of the psychological profile belong to different modalities, exhibiting differences in feature distributions and representation dimensions. In this study, a cross-modal attention mechanism is employed to achieve precise alignment and selective integration of the dual-modality features through adaptive weighting, while a gated fusion module dynamically regulates the contribution of each modality (26). Together, these components enable deep integration of heterogeneous modality information, providing robust cross-modal feature representations for health risk label representation.

Let the EEG spatiotemporal fusion feature sequence be $S=\left[s_{1},s_{2},\ldots ,s_{T}\right]$, and the psychological profile features constructed from the Big Five traits be *p*, which are encoded via an MLP. First, a linear transformation is applied to align the feature dimensions of the two modalities, eliminating the heterogeneity in representation dimensions (Equation 7):

(7) S′=S·We+bep′=ReLU(p·Wp+bp)

Where W*_e_* and W*_p_* are the weight matrices for the corresponding linear transformations, b*_e_* and b*_p_* are the corresponding bias vectors, S′ denotes the aligned EEG features, and *p*′ denotes the aligned psychological profile features.

Next, the psychological profile features are used as the query, and the EEG spatiotemporal fusion features serve as the key and value, forming a personality-guided cross-modal attention mechanism. This enables the psychological traits to adaptively focus on the critical temporal segments of the EEG signals. The core computations are as follows (Equation 8):

(8) $$\begin{array}{l} A=\frac{p^{\prime }\cdot S^{\prime T}}{\sqrt{d_{m}}}\\ \alpha =Soft\max(A)\\ e=\alpha \cdot S^{\prime } \end{array}$$

Where A denotes the attention score matrix, $\sqrt{d_{m}}$ is the scaling factor to mitigate the dimensionality explosion problem, a represents the normalized cross-modal attention weights, and e is the attention-weighted EEG fusion feature.

To dynamically fuse the attention-weighted EEG feature with the dimension-aligned psychological profile features, the following operation is performed (Equation 9):

(9) $$\begin{array}{l} g=\sigma \left(W_{g}\cdot [e;p^{\prime }]+b_{g}\right)\\ f=g\circ e+(1-g)\circ p^{\prime } \end{array}$$

Where W*_g_* denotes the gating weight matrix, b*_g_* is the gating bias term, g represents the gating weight, and ∘ is the Hadamard (element-wise) product.

During the cross-modal attention and gated fusion process, the gating weight g dynamically regulates the contributions of the dual-modality features. When g*_i_* → 1, the *i*-th dimension is also dominated by the EEG feature; and when g*_i_* → 0, it is dominated by the psychological profile feature. The final output *f* represents the deeply fused cross-modal feature, encompassing both the EEG neurophysiological representation and the psychological profile characteristics.

### 2.4 Design of cross modal temporal representation model

**Figure 1:**
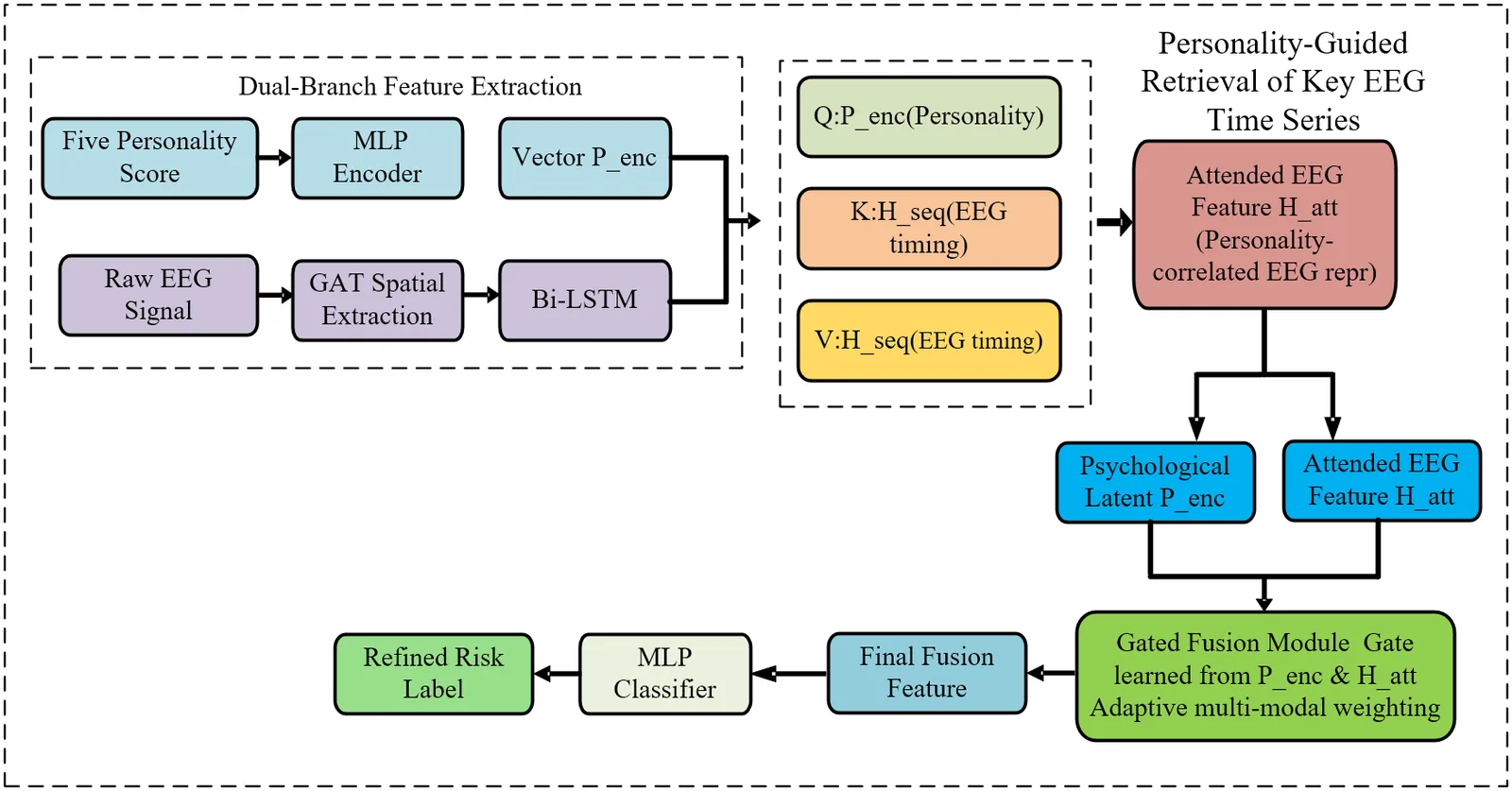
Cross-modal health risk assessment model integrating psychological profiles and EEG neurophysiological signals.

This study proposes a psychological-profile-guided cross-modal attention and gated fusion model for health risk assessment, termed PAG-Net (Psych-guided Attention & Gated fusion Network), as illustrated in **Figure 1**. The overall architecture adopts a dual-branch feature extraction strategy to separately model static psychological priors and dynamic EEG temporal features.

In the static modality branch, psychological profiles are constructed from the native Big Five personality scores and encoded via an MLP to map them into high-dimensional latent vectors, representing stable individual psychological traits. In the dynamic modality branch, the 14-channel EEG signals are first processed through a GAT to capture spatial topological relationships and dependencies among brain regions. Subsequently, a Bi-LSTM is introduced for bidirectional temporal modeling of the EEG sequence, producing EEG feature representations that incorporate contextual temporal information.

On this basis, the model incorporates a cross-modal interaction and adaptive fusion mechanism as its core module. In the cross-modal attention layer, the psychological profile features serve as query vectors to guide the retrieval of EEG features, adaptively weighting EEG temporal segments that are highly relevant to the current psychological state, resulting in attention-enhanced EEG representations. Then, the latent psychological vectors and attention-weighted EEG features are fed into a gated fusion module, which dynamically learns the contribution weights of both modalities in decision-making to form the final fused feature. This feature is subsequently input to an MLP classifier to output the corrected health risk labels.

The proposed architecture not only mitigates the subjectivity inherent in questionnaire-based assessments but also enhances the stability and interpretability of risk predictions, providing reliable support for personalized psychological interventions.

**Table 1** summarizes the key parameter configurations and training settings of the proposed model. The EEG spatiotemporal feature extraction module consists of a graph attention network (GAT) layer and a bidirectional long short-term memory (Bi-LSTM) layer. The GAT layer performs spatial feature mapping on the 14-channel EEG signals, with an output dimension of 64, and employs the LeakyReLU activation function to enhance nonlinear representation capability. When constructing the GAT adjacency matrix, Gaussian kernels are utilized to calculate the connectivity weights between EEG channels. The Gaussian kernel bandwidth is set to 0.8, and the distance threshold is set to 0.3. Connections between nodes are removed by assigning zero weights when the Euclidean distance between channels exceeds the predefined threshold.

**Table 1:** Parameter settings for the model in this article.

Module	Layer	Parameter	Setting
EEG Spatial Encoder	Input Nodes	Channel Number	14 EEG channels
	GAT Linear Mapping	Output Dimension	64
	Attention Head	Number of Heads	1
	Activation	LeakyReLU Slope	0.2
EEG Temporal Encoder	Bi-LSTM	Hidden Units	128
	LSTM Layers	Number of Layers	1
	Direction	Bidirectional	TRUE
	Output Dimension	Feature Size	256
Psychological Encoder	Input	Big Five Dimension	5
	MLP Hidden Layer	Hidden Units	64
	Output Layer	Portrait Dimension	128
Cross-Modal Attention	Query	Source	Psychological embedding P_enc
	Key/Value	Source	EEG temporal feature H_seq
	Attention Type	Similarity Function	Scaled Dot-Product
	Projection Dim	Feature Dimension	256
Gated Fusion	Gate Function	Activation	Sigmoid
	Fusion Strategy	Weighting Mechanism	Adaptive weighting
	Output Dimension	Feature Size	256
Classifier	Fully Connected	Hidden Units	128
	Output Layer	Class Number	3
	Activation	Function	Softmax
	Dropout	Rate	0.5

The Bi-LSTM layer is designed to capture temporal dependencies in EEG signals. Each directional LSTM contains 128 hidden units, and the forward and backward outputs are concatenated to form a 256-dimensional temporal representation.

The psychological profile encoding module maps the 5-dimensional Big Five personality features into a 128-dimensional high-level latent representation through a two-layer multilayer perceptron (MLP), which is subsequently used to guide cross-modal attention interaction. In the cross-modal attention mechanism, the psychological profile representation is employed as the Query, while EEG temporal features are used as the Key and Value. Scaled dot-product attention is adopted to calculate attention weights, with the projection dimension set to 256, enabling the model to focus on EEG patterns highly associated with personality characteristics.

The gated fusion module dynamically adjusts the contribution weights of psychological profile features and EEG representations in the final fused representation through a Sigmoid activation function. The classifier consists of a fully connected layer with 128 hidden units, followed by a Softmax layer for final prediction.

In all experiments, a subject-independent data partitioning strategy was adopted to strictly prevent data leakage caused by samples from the same participant appearing across the training and testing sets.

## 3 Experimental design and verification protocol

### 3.1 Dataset and preprocessing

This study utilizes the AMIGOS dataset (A Multimodal Dataset for Affect Recognition Using Psychological and Physiological Signals) as the experimental data source. The AMIGOS dataset is designed to provide high-quality, multidimensional annotations for research on multimodal emotion recognition, personality trait associations, and mental health state assessment. It synchronously collects participants’ psychological questionnaire scores, self-reported emotional states, and multichannel physiological signals. The dataset is complete, reliable, and highly compatible with the proposed cross-modal health risk assessment task combining psychological profiles and EEG spatiotemporal features.

#### 3.1.1 Dataset modality and composition

**Figure 2:**
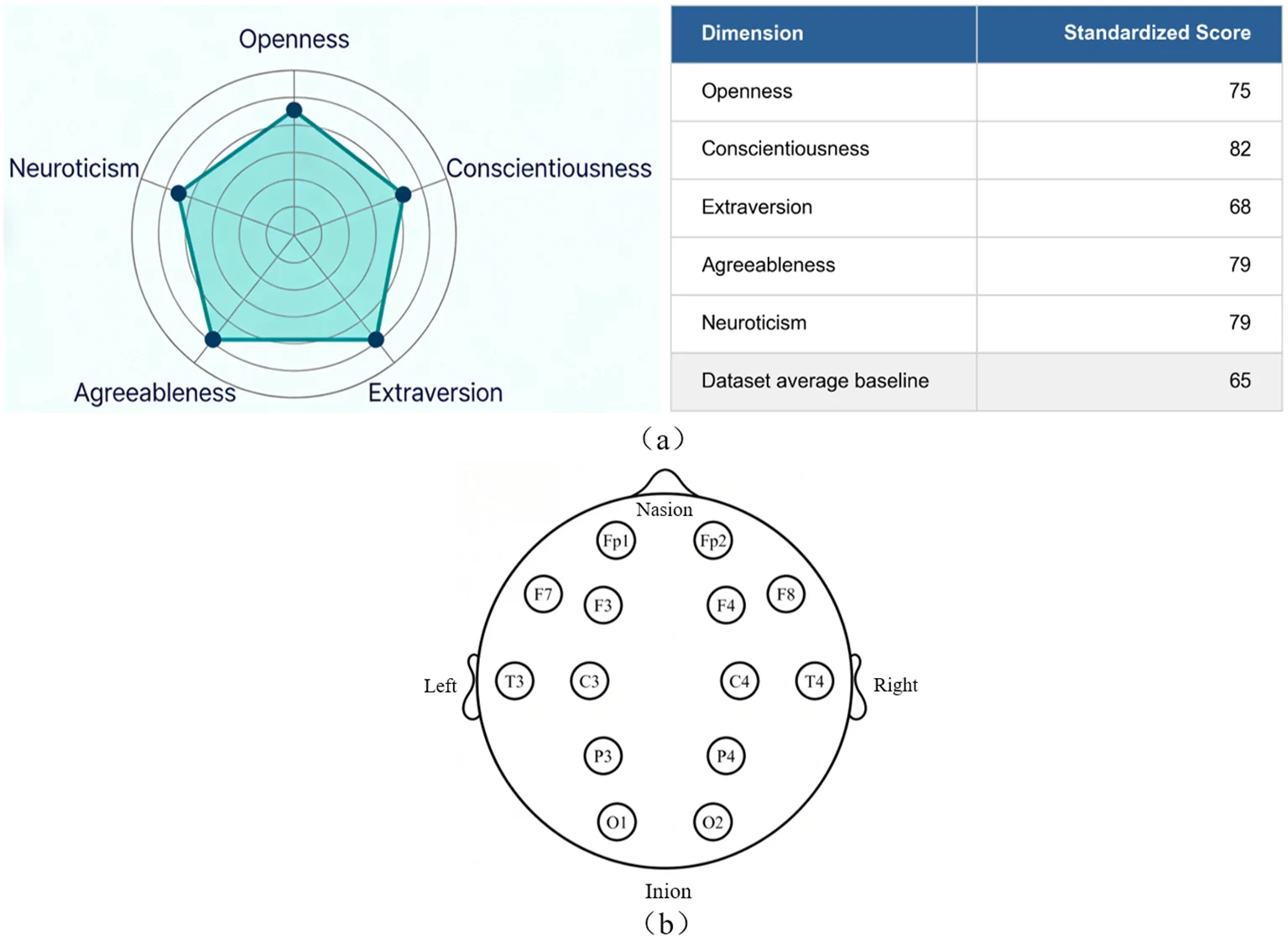
Big Five personality scores and distribution of the 14 EEG electrodes. **(a)** Radar chart displaying the five personality traits (openness, conscientiousness, extraversion, agreeableness, and neuroticism) with standardized scores. **(b)** Diagram of a top view of a human head illustrating the 14 EEG electrode positions.

The AMIGOS dataset contains two types of data modalities—psychological and physiological—supporting the static psychological feature branch and the dynamic EEG branch in this study. The psychological modality data are derived from the dataset’s native standardized assessments, including Big Five personality scores and self-reported emotional ratings. The Big Five Personality Model provides standardized scores for each participant across five dimensions: Openness, Conscientiousness, Extraversion, Agreeableness, and Neuroticism. These five-dimensional raw scores are used to construct individual psychological profiles, as illustrated in **Figure 2a**. For each personality dimension, the corresponding questionnaire items were first aggregated to obtain the raw score, after which min–max normalization was independently applied to map the scores into the range of [0,1]. Each personality dimension was normalized separately, without summation or joint normalization across different dimensions. For visualization, the normalized scores were linearly rescaled to 0–100 in **Figure 2a**. The bottom value of 65 represents the global mean of the standardized personality scores across all participants in the AMIGOS dataset and serves only as a reference baseline for comparison; it is not derived from the five personality scores of the displayed participant nor used in the subsequent health risk score calculation. Considering the established association between Neuroticism and mental health risk, the normalized Neuroticism score was combined with the corresponding Valence and Arousal ratings of each video to construct the continuous health risk score.

The physiological modality in this study primarily consists of EEG signals. The dataset was collected using a standard multi-channel EEG acquisition system with 14 EEG channels. The electrode configuration was selected as a subset of the international 10–20 system, including Fp1, Fp2, F3, F4, C3, C4, P3, P4, O1, O2, F7, F8, T3, and T4. The EEG signals were recorded at a sampling rate of 250 Hz, as illustrated in **Figure 2b**.

#### 3.1.2 Data selection plan

The AMIGOS dataset is designed based on an emotion induction paradigm, consisting of short-video and long-video experiments, with 40 and 37 valid participants, respectively. During each experiment, participants were required to provide subjective ratings on a 9-point Likert scale after viewing each video, including valence, arousal, dominance, familiarity, and liking. Among these, valence and arousal serve as core indicators of transient affective states and, together with the Big Five personality scores, form the initial health risk labels in this study.

Considering that participants’ states are more stable and signal artifacts are fewer in the short-video paradigm, and that the sample size is suitable for model training, this study selected the short-video subset as the research focus. Multimodal psychological and EEG data from 20 participants were used, and samples with severe signal loss or abnormal ratings were excluded. The resulting high-quality paired psychological-physiological dataset was used for training and validating the proposed cross-modal health risk assessment model.

#### 3.1.3 Data preprocessing

A standardized multi-stage preprocessing pipeline was designed for the 14-channel raw EEG signals. First, baseline correction was performed to eliminate direct current offsets and low-frequency drifts. Subsequently, a 0.5–50 Hz Butterworth band-pass filter was applied to preserve EEG frequency components associated with emotional and psychological states. Independent component analysis (ICA) was then employed to remove physiological artifacts, including electrooculographic (EOG) and electromyographic (EMG) contamination, while abnormal fluctuation segments were excluded. The ICA components were identified using a semi-automatic strategy combining automated classification and expert verification. Specifically, the ICLabel model was first used to categorize independent components into EOG-, EMG-, and brain-related sources, followed by manual inspection of ambiguous components by two researchers with EEG analysis experience to improve artifact identification reliability.

Considering the experimental paradigm, each participant completed 16 independent emotion-inducing video sessions, which were continuously recorded during EEG acquisition. During preprocessing, video timestamps were used as segmentation boundaries, and the continuous EEG recordings were divided into 16 non-overlapping single-video EEG segments. Signal concatenation across different videos was strictly avoided. Sliding-window segmentation was performed only within each individual video segment, where each window contained 128 sampling points, corresponding to a 0.512-s EEG sequence under the sampling rate of 250 Hz. After processing all video segments, each participant generated a total of 1,378 temporal EEG samples, with each sample represented as a matrix of 14 × 128 (channels × time points). All window samples within the same video segment were assigned the corresponding self-reported affective scores of that video, ensuring accurate one-to-one alignment between EEG signals and synchronized psychological annotations.

Based on the normalized psychological features, a continuous health risk score was constructed by integrating stable personality traits and video-induced transient emotional responses. Specifically, the neuroticism dimension of the Big Five personality model was combined with the instantaneous valence and arousal ratings elicited by each video, and an equal-weighted formulation was adopted to calculate the composite risk score. For psychological data processing, the original Big Five personality scores and affective ratings, including valence and arousal, were first normalized into a unified scale. Since all scores were collected using a 9-point Likert scale, linear normalization was applied to transform the original values x∈ [1,9] into the range of [0,1] to eliminate scale differences, as follows (Equation 10):

(10) $$x^{\prime }=\frac{x-1}{8}$$

where *x*′ denotes the normalized psychological feature value.

Based on the normalized neuroticism, arousal, and valence features, an individual continuous health risk score *R* was constructed, which is defined as follows (Equation 11):

(11) $$R=w_{1}N+w_{2}A+w_{3}(1-V)$$

where *N*, *A*, *V* represent the normalized psychological features, and 1-*V* is used to characterize the intensity of negative emotional states. Equal weights are assigned to each feature as follows (Equation 12):

(12) $$w_{1}=w_{2}=w_{3}=\frac{1}{3}$$

Furthermore, to construct supervised learning labels, the continuous risk scores *R* were categorized into three risk levels (low, medium, and high) using a quantile-based partitioning strategy based on the distribution of *R* in the training set. Specifically, the low-, medium-, and high-risk groups were defined according to *R* ≤ *P*_33_, *P*_33_< *R*< *P*_66_ and *R* > *P*_66_, respectively, where *P*_33_ and *P*_66_ denote the 33rd and 66th percentiles of the risk score distribution.

The proposed model simultaneously takes static personality representations and temporal EEG features as inputs, integrating long-term psychological traits with video-induced short-term emotional neural responses. This design enables cross-individual psychological risk representation learning by jointly modeling heterogeneous psychological and physiological information, rather than focusing solely on transient emotional states or static personality characteristics.

**Figure 3:**
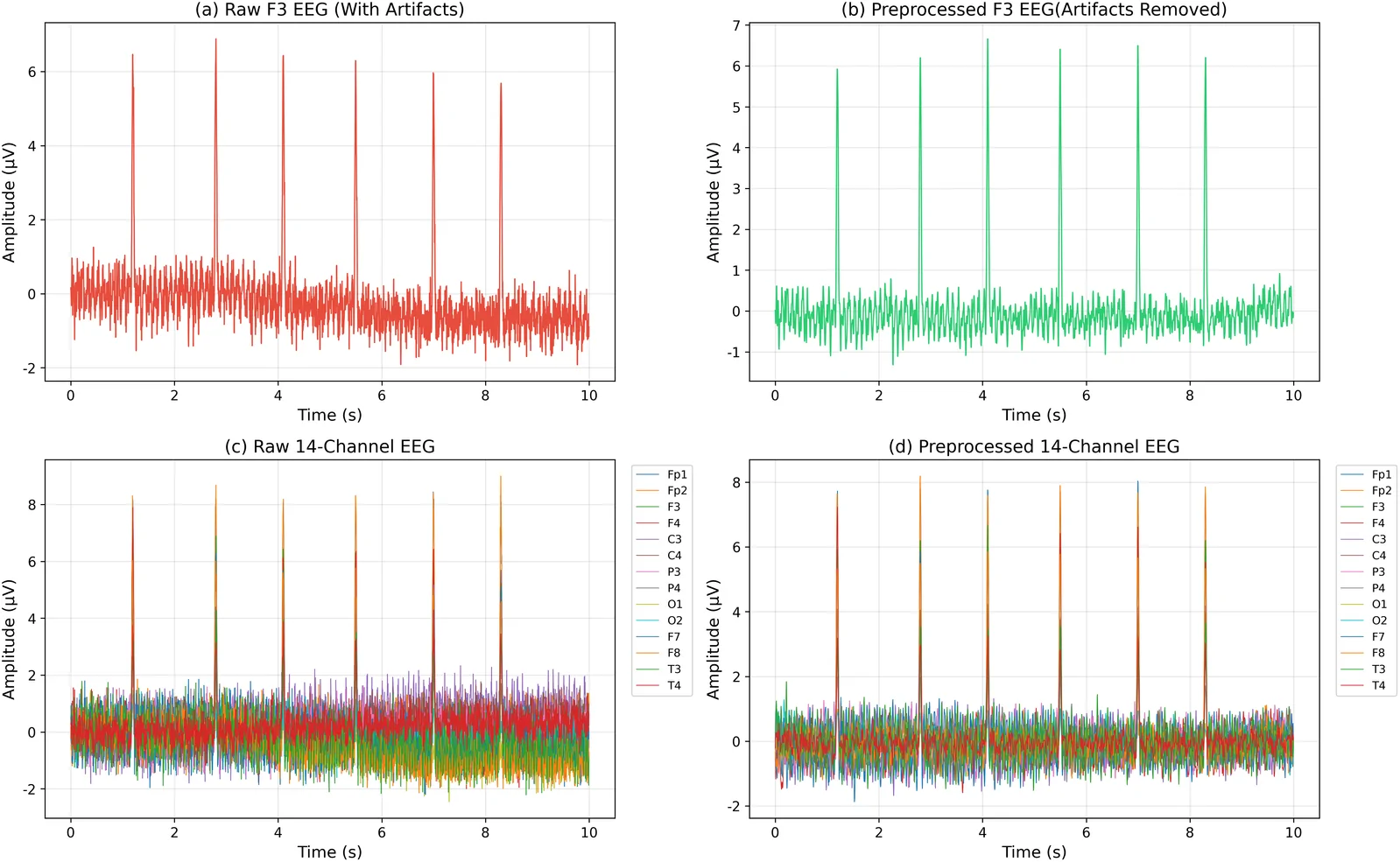
Time-domain comparison of EEG signals before and after preprocessing. **(a)** Raw F3 channel EEG with artifacts. **(b)** Preprocessed F3 EEG with reduced artifacts. **(c)** Raw EEG from fourteen channels with overlapping traces. **(d)** Preprocessed fourteen-channel EEG with cleaner signals.

**Figure 4:**
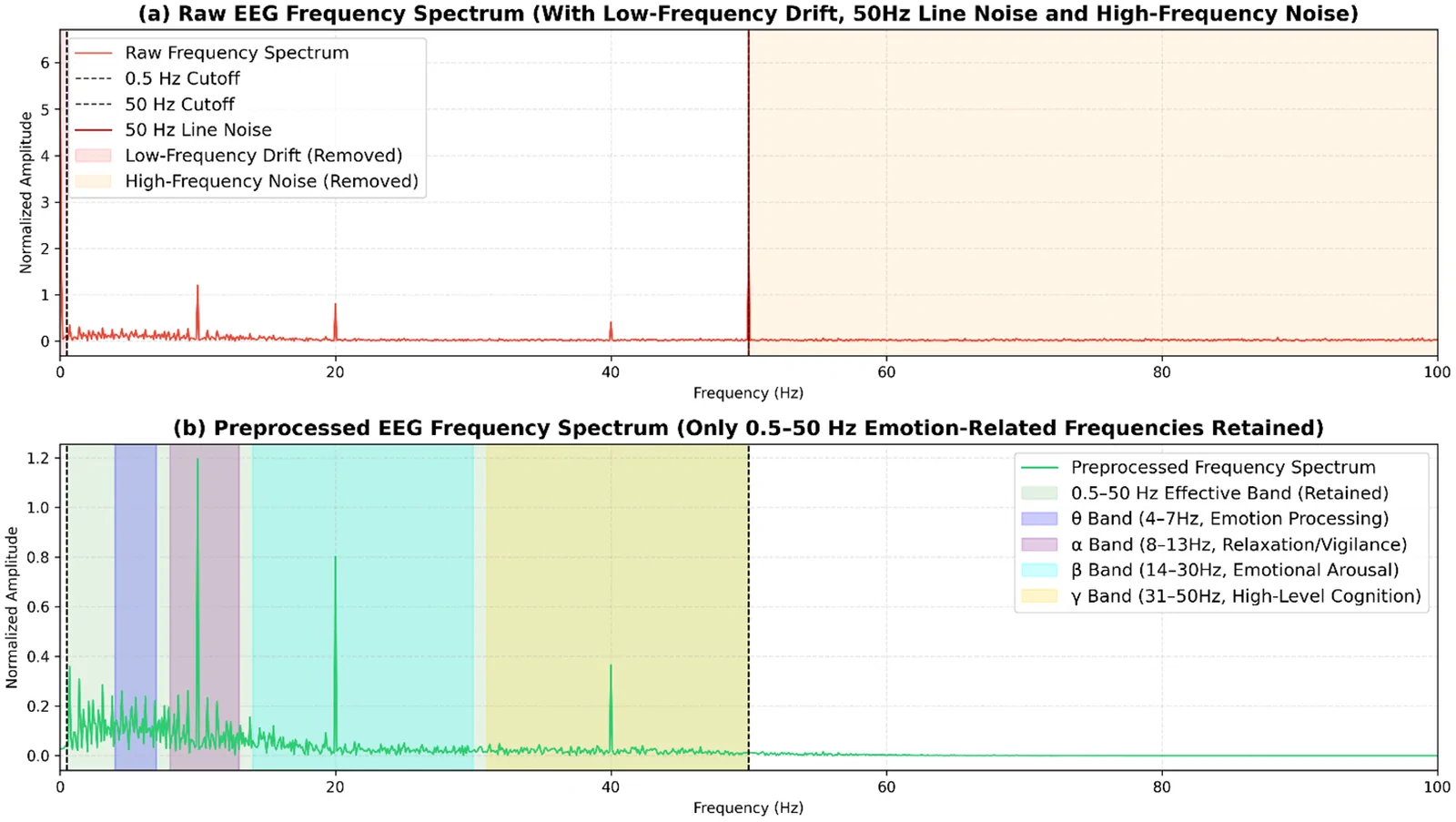
Frequency-domain distribution of EEG signals before and after preprocessing. **(a)** Raw EEG frequency spectrum with low-frequency drift and high-frequency noise shaded, illustrating artifacts before filtering. **(b)** Preprocessed EEG spectrum, retaining only 0.5–50Hz emotion-related bands, with color-coded theta, alpha, beta, and gamma bands highlighted.

**Figure 3** presents a representative time-domain EEG waveform from the F3 channel of a typical participant, demonstrating that baseline drift and physiological artifacts were effectively suppressed after ICA-based artifact removal. **Figure 4** illustrates the spectral comparison before and after preprocessing, confirming that the 0.5–50 Hz band-pass filtering procedure preserved emotion-related EEG frequency components while attenuating low-frequency drifts and high-frequency noise.

### 3.2 Experimental environment setup

The experiments were conducted on an Ubuntu 20.04 LTS operating system, with model development implemented using Python 3.8 and the PyTorch 1.10 deep learning framework. Data processing and visualization relied on NumPy, Pandas, Matplotlib, and Seaborn libraries. The hardware setup consisted of an Intel Core i7 processor with 16 GB DDR4 memory, and an NVIDIA GeForce RTX 3060 GPU (12 GB VRAM) was employed to accelerate training.

For model training, the Adam optimizer was used with an initial learning rate of 0.001 and a weight decay of 1e-4. The batch size was set to 32, and the model was trained for 50 epochs to ensure convergence while mitigating overfitting.

Since the temporal EEG samples generated by sliding-window segmentation from the same participant exhibit strong intra-subject correlations, they cannot be regarded as independent samples. Randomly partitioning these samples may lead to data leakage, resulting in overoptimistic performance estimates and compromised generalization evaluation. To address this issue, a subject-independent partitioning strategy was adopted. Specifically, complete participants, rather than individual EEG segments, were used as the basic partitioning unit, and the dataset was divided into training, validation, and testing sets in a ratio of 7:1:2. All temporal EEG samples belonging to a given participant were assigned exclusively to a single subset, ensuring that no participant appeared in more than one subset.

Model parameters were optimized using data from 14 participants in the training set, while the 4 participants in the test set remained completely unseen throughout both model training and hyperparameter tuning. This protocol effectively eliminates the influence of intra-subject signal correlations on performance evaluation and provides a more reliable assessment of the model’s cross-subject generalization capability. Furthermore, all baseline methods and ablation studies employed the identical subject-independent partitioning protocol to ensure a fair and consistent experimental comparison.

## 4 Results and discussion

### 4.1 Comparison of different modes

To systematically evaluate the effectiveness of the proposed cross-modal fusion model, four comparative approaches were constructed, progressing from single-modality to dual-modality models and from simple fusion to deep fusion, forming a hierarchical validation framework. The structural configurations and design objectives of these models are summarized in **Table 2**.

**Table 2:** Structural configurations and design objectives of different modalities.

Model	Input modality	Feature encoding	Fusion strategy	Design objective
Psychologyonly	Big Five persona- lity features	Two-layer MLP encoding of psychological vector	No fusion (single-modality)	Evaluate baseline performance of traditional psychological assessments in health risk prediction without EEG information
EEG only	EEG temporal Signals	GAT for spatial channel relations + Bi-LSTM for temporal dependencies	No fusion (single-modality)	Assess the discriminative capability of EEG signals for health risk recognition
Simple Fusion	EEG + psycholo- gical features	EEG: GAT+Bi-LSTM; Psychology: MLP	Direct concatenation into MLP classifier	Validate the effect of naive dual-modality fusion based solely on feature concatenation
PAG-Net	EEG+psycholo- gical features	EEG: GAT+Bi-LSTM; Psychology: MLP	Cross-modal attention guidance gated fusion with dynamic weighting	Leverage psychological vectors to guide EEG feature selection, enabling adaptive correction of initial psychological labels

First, a psychology-only baseline model was established, in which a two-layer MLP encoder was applied to the standardized Big Five personality features to directly predict health risk labels. This model serves to characterize the baseline performance of traditional single-modality psychological assessments on this task.

Second, an EEG-only model was constructed, utilizing the GAT and Bi-LSTM combination to extract spatial correlations and temporal dependencies from raw EEG signals. This allows evaluation of the independent discriminative capability of EEG features for health risk prediction.

Third, a simple dual-modality concatenation model was designed, in which EEG spatiotemporal features and psychological latent vectors were directly concatenated and fed into an MLP classifier, without incorporating cross-modal attention or gated fusion mechanisms. This model evaluates the performance gain brought solely by combining dual-modality information.

Finally, the proposed cross-modal attention and gated fusion model was implemented, where psychological latent vectors guide EEG feature selection, and a gated mechanism dynamically allocates contribution weights between the two modalities, enabling adaptive correction of the initial psychological risk labels.

Through these comparative settings, the individual contributions of the psychological modality, EEG modality, simple fusion, and the proposed deep fusion strategy can be systematically analyzed, highlighting their respective roles and performance advantages in the health risk assessment task.

To systematically evaluate the effectiveness of the proposed model, PAG-Net was compared with three baseline models in a cross-subject three-class psychological health risk prediction task. Performance was assessed using four core metrics: accuracy, weighted F1 score, macro F1 score, and AUC. Accuracy reflects the overall proportion of correctly classified samples, weighted F1 accounts for class imbalance by weighting each class according to sample size, macro F1 treats all classes equally to capture overall precision and recall performance, and AUC (One-vs-Rest) evaluates the model’s discriminative ability across the three risk levels at the probability level.

**Figure 5:**
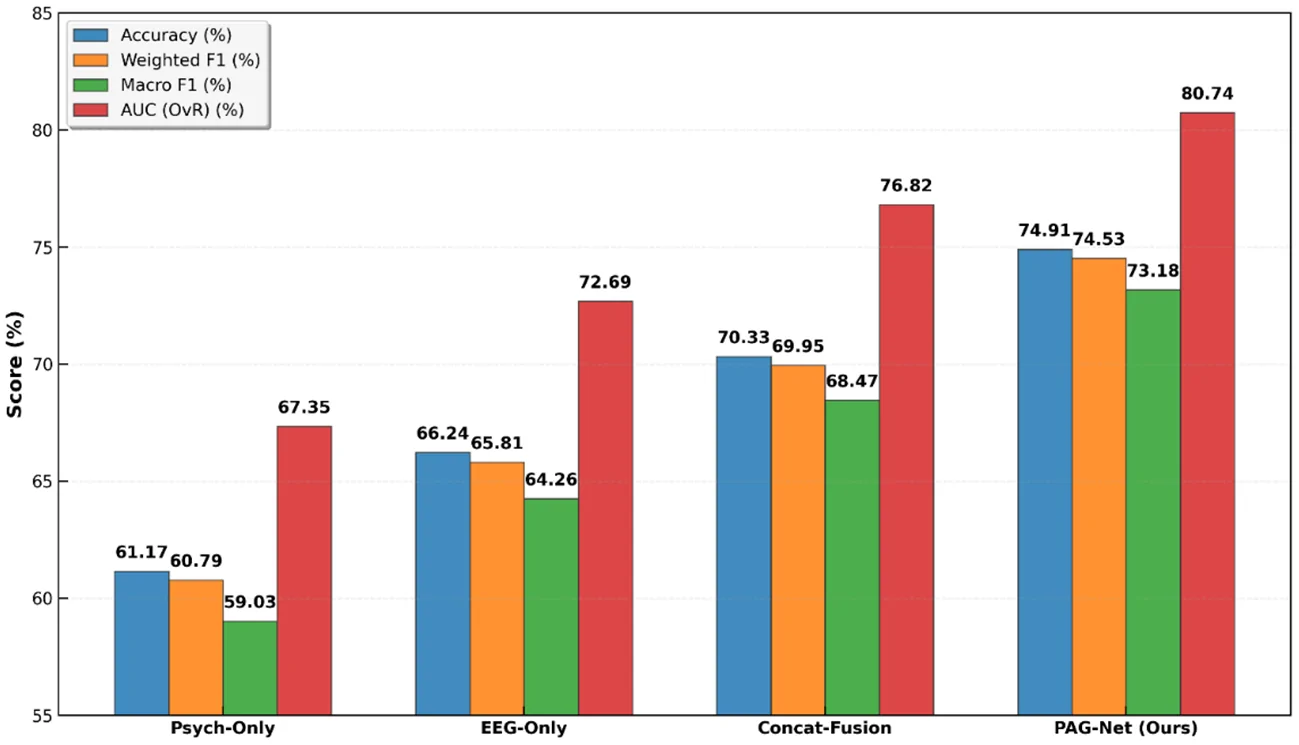
Performance comparison of different models.

The results, as shown in **Figure 5**, reveal a clear progressive improvement in performance with increasing depth of modality fusion. The EEG-only model outperformed the psychology-only baseline, indicating that EEG signals effectively capture physiological features related to psychological health risk. The simple dual-modality concatenation model further improved performance over the single-modality models, demonstrating the baseline benefits of integrating psychological and EEG information. Notably, the proposed PAG-Net cross-modal fusion model achieved the highest performance across all metrics, with accuracy, weighted F1, macro F1, and AUC reaching 74.91%, 74.53%, 73.18%, and 80.74%, respectively—showing improvements of over 4% compared with the simple concatenation model.

These results substantiate that the proposed cross-modal fusion strategy can more effectively leverage the complementary information between psychological traits and EEG features, enabling deep integration and synergistic optimization of dual-modality data, and thereby significantly enhancing the accuracy and reliability of cross-subject psychological health risk assessment.

#### 4.1.2 Comparison of initial and multimodal risk representations

**Figure 6:**
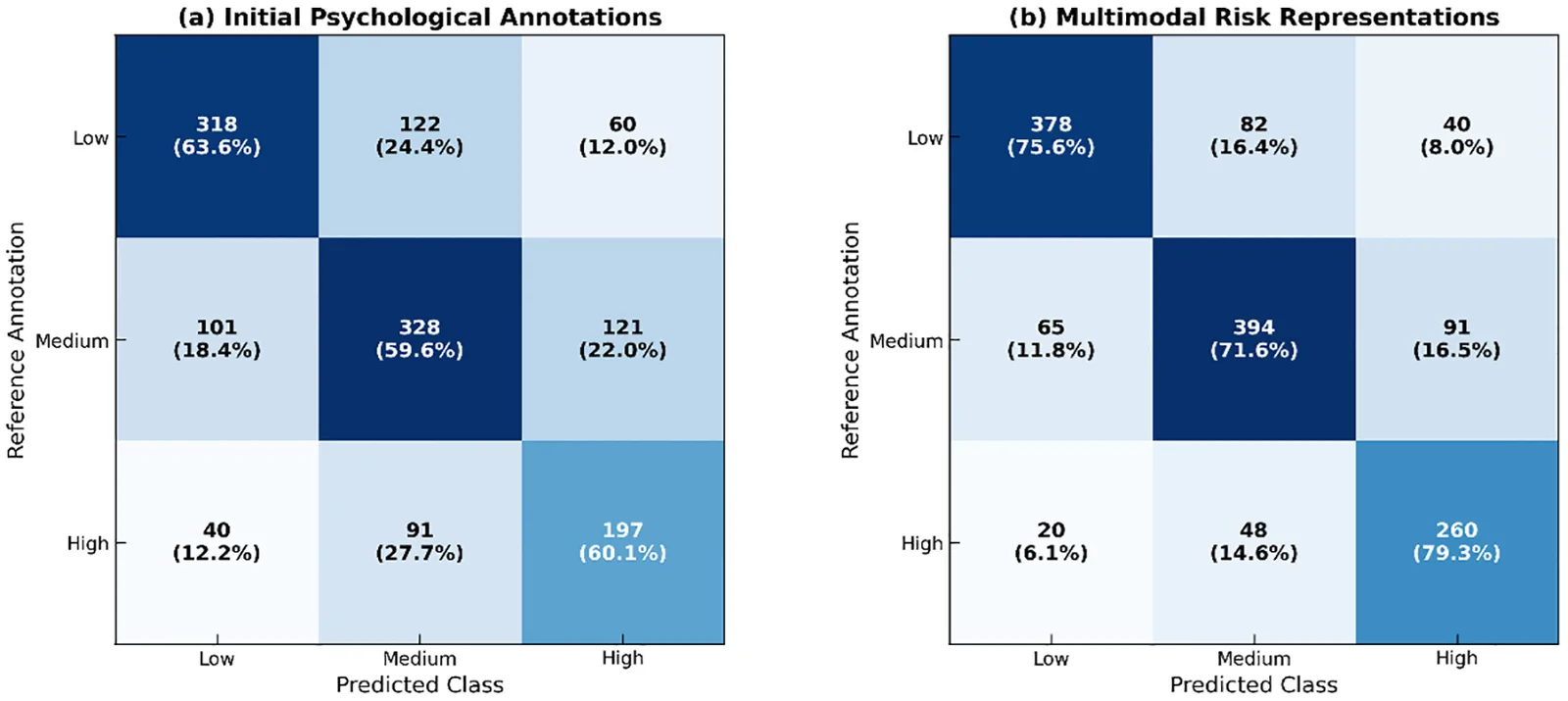
Confusion matrices of the initial and multimodal risk representations. **(a)** Confusion matrix using the initial psychological annotations as the reference. **(b)** Confusion matrix using the multimodal risk representations generated by PAG-Net as the reference.

To further investigate the influence of EEG information on psychological risk representation, a single test participant was selected for case analysis. Specifically, the 1,378 temporal EEG samples generated from this participant were analyzed to compare the initial risk representations constructed from psychological features and the multimodal risk representations generated by PAG-Net in a three-class classification task, as illustrated in **Figure 6**. This analysis was performed as an illustrative case study to visualize the changes in risk representation after incorporating EEG information, rather than as an evaluation of the overall model performance. Specifically, **Figure 6a** uses the initial psychological annotations as the reference annotations, whereas **Figure 6b** uses the multimodal risk representations generated by PAG-Net as the reference annotations. The initial psychological annotations were constructed from the Big Five personality traits and self-reported affective ratings, representing conventional risk stratification based solely on psychological information. In contrast, the multimodal risk representations were obtained by integrating EEG spatiotemporal features with latent psychological representations, enabling a cross-modal refinement of the original risk representations.

A comparison between **Figures 6a, b** shows a noticeable improvement in the consistency of sample assignments after incorporating EEG information. The proportion of samples located along the main diagonal increases, indicating higher agreement between the model outputs and the corresponding reference annotations. In particular, the medium-risk category exhibits a more balanced sample distribution, with fewer samples concentrated in the high-risk category. These findings suggest that EEG-derived physiological information provides complementary information to questionnaire-based psychological assessments and influences the distribution of psychological risk representations.

Quantitatively, the agreement between the model outputs and the corresponding reference annotations increases from 61.17% under the initial psychological annotation scheme to 74.91% under the multimodal risk representation scheme. It should be emphasized that this metric reflects the consistency between the model outputs and the corresponding reference annotations, rather than an improvement in absolute prediction accuracy.

It should also be noted that, in the absence of independent external ground truth, such as clinical diagnoses or structured psychiatric assessments, both the initial psychological annotations and the multimodal risk representations are data-driven representations of psychological risk. Therefore, the comparison in **Figure 6** is intended only to illustrate the differences and consistency between the two representation schemes, rather than to demonstrate that either representation more accurately reflects the participants’ true mental health status.

#### 4.1.3 Ablation experiment

**Figure 7:**
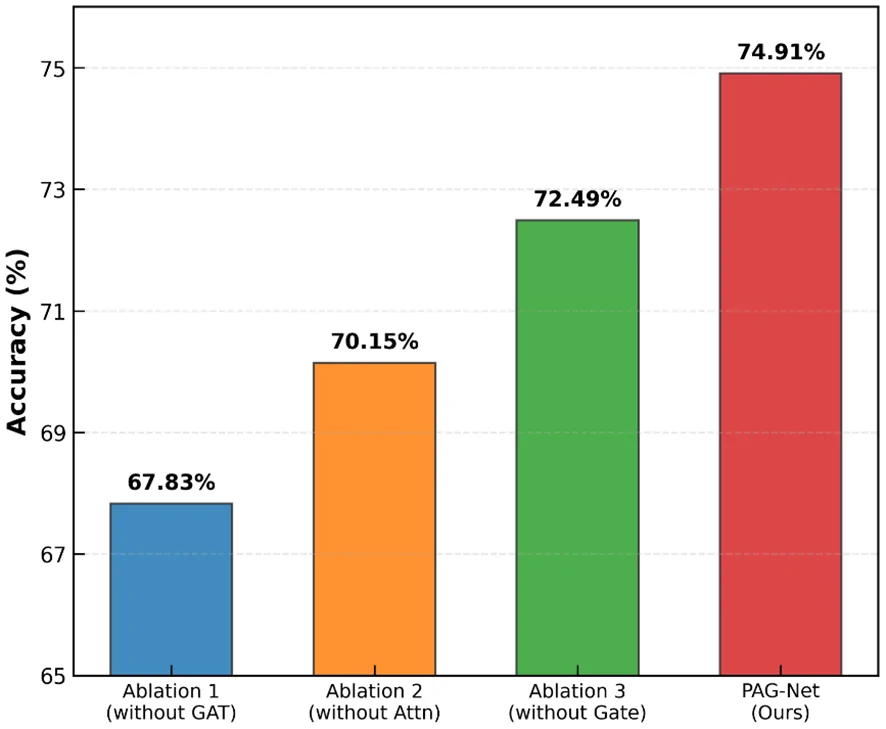
Ablation study accuracy comparison.

To quantify the contribution of each core component, an ablation study was conducted using classification accuracy as the primary metric, and the results are reported in **Figure 7**. The three ablated variants exhibit a stepwise recovery pattern, where performance degradation decreases progressively as modules are restored.

Specifically, removing the GAT-based spatial modeling module reduces the accuracy to 67.83%, which corresponds to a drop of 7.08% compared with the complete model. This is the largest decline among all ablation settings, indicating that graph-structured EEG channel interaction modeling forms the foundation for discriminative representation learning. Without explicitly capturing inter-electrode dependencies, the model loses substantial spatial contextual information.

When the personality-guided cross-modal attention mechanism is excluded, the accuracy drops to 70.15%, which corresponds to a drop of 4.76% compared with the complete model. This observation suggests that psychological-profile-driven alignment and selective emphasis help the network concentrate on EEG segments that are more consistent with individual traits, thereby improving the utilization efficiency of heterogeneous information.

Further removing the gated fusion module yields an accuracy of 72.49%, with only a 2.42 percentage point decrease relative to the full architecture. This indicates that dynamic gating mainly serves as a high-level refinement layer, enabling fine-grained complementarity between modalities once reliable representations have been formed.

Finally, the complete PAG-Net achieves the best performance with an accuracy of 74.91%, which verifies the independent effectiveness and hierarchical synergy of GAT-based spatial modeling, cross-modal attention guidance, and adaptive gated fusion in boosting psychological health risk assessment.

#### 4.1.4 Quantitative analysis of attention weights and personalized contribution intervention

To further investigate the functional role of EEG features during cross-modal fusion and to interpret how critical neural patterns contribute to psychological health risk prediction, a quantitative analysis was conducted on the output weights of the cross-modal attention layer in PAG-Net. Specifically, the trained model was applied to the test set, and the attention weight matrices assigned to EEG representations were extracted from the personality-guided cross-modal attention module.

**Figure 8:**
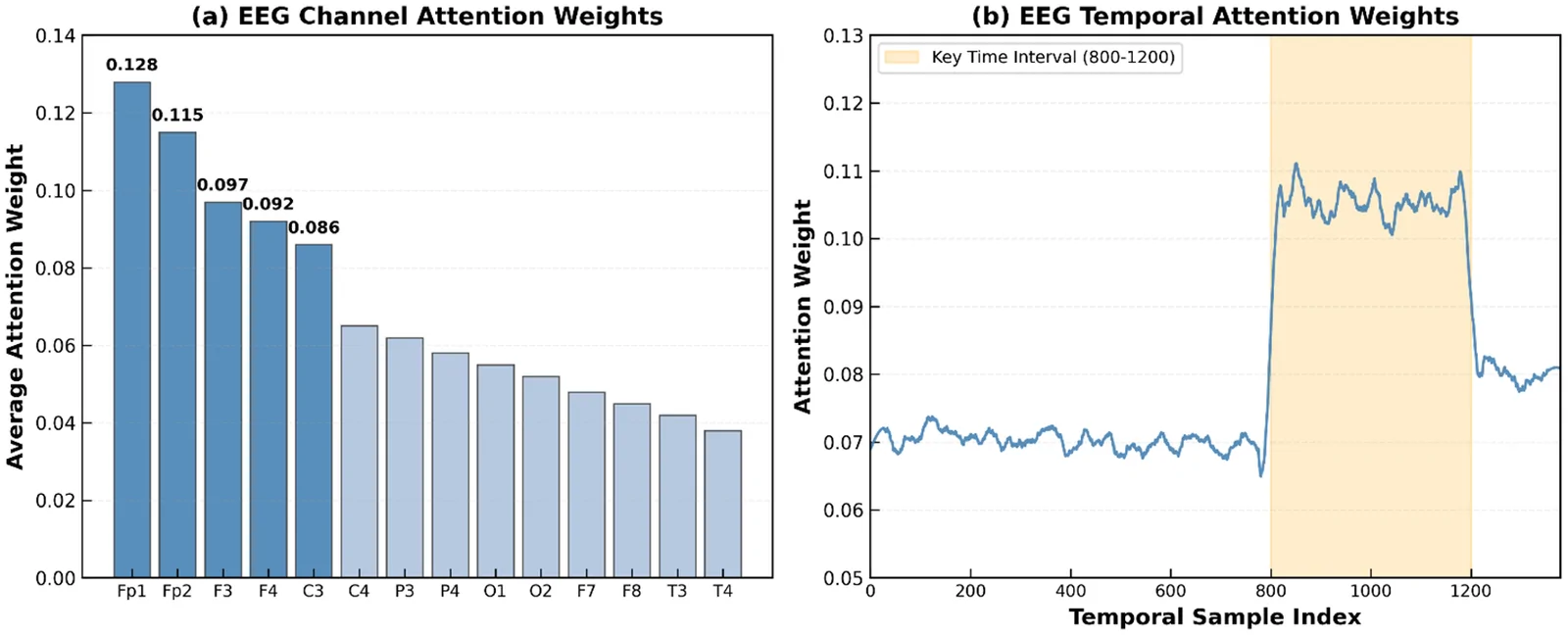
Attention weight quantification analysis. **(a)** Average EEG channel attention weights across the 14 channels, highlighting the Top-5 contributing electrodes (Fp1, Fp2, F3, F4, and C3). **(b)** EEG temporal attention weights over temporal sample indices, highlighting key time intervals (samples 800–1200).

The analysis was performed along two dimensions: the channel dimension and the temporal dimension. The channel dimension corresponds to the 14 EEG electrodes and reflects the relative importance of different brain regions in the model’s decision process, while the temporal dimension corresponds to time segments within each EEG sample and characterizes the influence of different moments on risk discrimination. By computing the mean attention weights and their distributions across samples, channels and temporal segments were ranked to identify the most informative EEG components. As a result, the Top-5 contributing EEG channels (**Figure 8a**) and Top-10 key temporal segments (**Figure 8b**) were selected, enabling preliminary localization of critical neural features. Furthermore, attention distributions across different risk levels were compared to explore the association between EEG patterns and psychological health risk, providing physiological references for personalized intervention strategies.

As shown in **Figure 8a**, the ranking of average attention weights across the 14 EEG channels indicates that Fp1, Fp2, F3, F4, and C3 are the most influential channels (Top-5), with mean weights of 0.128, 0.115, 0.097, 0.092, and 0.086, respectively, which are markedly higher than those of the remaining channels (mean< 0.07). From a neurophysiological perspective, these electrodes mainly cover the prefrontal and central regions. The prefrontal cortex plays a central role in emotion regulation and cognitive control, while the central area is related to somatosensory processing and arousal modulation. These functional characteristics are highly consistent with mechanisms underlying psychological risk, such as emotional dysregulation and cognitive bias. The results indicate that the proposed model automatically captures EEG channels most strongly associated with mental health risk, validating the rationality of the personality-guided attention strategy.

**Figure 8b** presents the analysis of temporal attention distributions. The key temporal segments are mainly concentrated in the middle-to-late stage of the experiment (samples 800–1200), where the average attention weight (0.103) is significantly higher than that in the early stage (0.068) and the final stage (0.075). This suggests that the contribution of EEG features to risk prediction exhibits clear temporal dynamics. As the experiment progresses, subjects’ neural responses tend to stabilize, and EEG patterns related to psychological states become more salient. Through the attention mechanism, the model automatically focuses on these high-value temporal segments, which further enhances prediction reliability.

Moreover, comparing attention distributions across different risk groups reveals distinct individual-level patterns. For high-risk (High) subjects, the prefrontal channels Fp1 and Fp2 receive notably higher attention, with mean values of approximately 0.142, indicating that prefrontal-related functions play a dominant role in model inference. This finding implies that impaired emotion regulation capacity may be a key physiological characteristic of high-risk individuals. For medium-risk (Medium) subjects, the central channel C3 shows relatively higher importance, which may be associated with abnormal somatic arousal and its regulation. In contrast, low-risk (Low) subjects exhibit a more uniform distribution of attention weights without pronounced dominant channels, reflecting more stable neural patterns.

These differentiated contribution profiles provide objective guidance for personalized psychological intervention. For high-risk individuals, interventions may focus on prefrontal-related training, such as cognitive behavioral therapy or EEG biofeedback targeting emotion regulation. For medium-risk individuals, strategies emphasizing arousal modulation, such as relaxation training or breathing control, may be more suitable. In this way, PAG-Net supports a pipeline from feature localization to targeted intervention, enabling more precise and individualized mental health management.

## 5 Compared with existing related research

As shown in **Table 3**, existing studies can generally be categorized into two research directions: EEG-based unimodal analysis and psychological feature-based modeling. EEG-based approaches, such as SVM- and DNN/CNN-based models, primarily exploit time-frequency characteristics or deep feature representations of EEG signals for emotion recognition. Some studies further enhance feature representation through simple feature fusion strategies. However, due to the lack of high-level psychological semantic guidance, these methods mainly focus on neural signal pattern recognition and provide limited capability for modeling the relationship between physiological activity and individual psychological characteristics.

**Table 3:** Comparison of related studies.

Method	Input modality	Model characteristics	Fusion strategy	Task
SVM (27)	EEG	Unimodal machine learning	None	Emotion classification
DNN/CNN (28)	EEG	Unimodal deep learning	Early fusion	Emotion recognition
Questionnaire-based method (29)	Questionnaire data	Statistical learning	None	Psychological assessment
Proposed method	EEG + Psychological features	Cross-modal deep learning	Latent interaction	Psychological risk representation

Another line of research is based on psychological questionnaires, where personality traits and self-reported affective ratings are analyzed using statistical methods or machine learning techniques to assess psychological states. Although these approaches effectively characterize explicit psychological attributes, they rely predominantly on subjective self-reported information and may therefore be influenced by individual cognitive bias, social desirability, and the limited dimensionality of questionnaire-based assessments. As a result, they are less capable of capturing the underlying neurophysiological mechanisms associated with psychological states.

Compared with these unimodal approaches, the proposed method integrates EEG-derived physiological information with standardized psychological features to establish a cross-modal risk representation framework. By introducing a latent interaction mechanism, the proposed model explores the intrinsic relationships between psychological characteristics and EEG spatiotemporal patterns, enabling joint modeling of subjective psychological information and objective neural activity. Rather than focusing solely on emotion recognition or questionnaire-based psychological score prediction, this study investigates multimodal psychological risk representation, providing a new perspective for improving the completeness of information utilization and the generalization capability of psychological risk assessment.

Nevertheless, several limitations should be acknowledged. The current framework incorporates only EEG signals and psychological features, while other physiological modalities, such as heart rate and electrodermal activity, have not been considered. In addition, the model is evaluated on a relatively limited public dataset, and its generalization capability across larger and more diverse populations remains to be further validated. Future work will incorporate additional physiological modalities and further optimize the cross-modal interaction mechanism to improve the robustness and applicability of the proposed framework.

## 6 Limitations analysis

Although the proposed multimodal psychological risk representation framework effectively integrates EEG-derived physiological information with psychological features and demonstrates promising performance, several limitations should be acknowledged.

First, the proposed method was evaluated using the publicly available AMIGOS dataset, which is relatively limited in scale and participant diversity. Consequently, the generalization capability of the proposed model across larger, more diverse, and real-world populations remains to be further investigated. Future studies will incorporate additional public datasets and clinical data to conduct cross-dataset validation and further improve the robustness and generalizability of the proposed framework.

Second, this study lacks independent external ground truth, such as clinical diagnoses, structured psychiatric interviews, or other objective psychological assessment criteria. Therefore, both the initial psychological annotations and the multimodal risk representations are data-driven representations that characterize the risk distributions derived from a single psychological information source and a joint psychological–physiological information source, respectively, rather than the participants’ actual mental health status. The differences between these two representations primarily reflect the redistribution of the risk representation space after incorporating EEG-derived physiological information, rather than indicating that the multimodal representation is inherently closer to the true psychological risk. Treating the multimodal representation as the ground truth for evaluating the initial annotations may introduce confirmation bias and circular analysis, since the model would effectively be validated using its own generated outputs. Accordingly, this study considers the two representations as alternative risk characterization schemes and compares them only in terms of their consistency, without assuming that either representation is superior or closer to the true psychological condition.

Third, although ICA was applied for EEG artifact reduction, the relatively low-density EEG configuration in the AMIGOS dataset (14 channels) may limit the complete separation of neural and non-neural components. In this study, ICA was performed on continuous EEG recordings with sufficient temporal samples, and artifact components were identified using a combination of automated classification and expert verification. However, the performance of ICA can still be affected by channel density and signal characteristics. Future studies should investigate higher-density EEG systems or integrate additional artifact correction strategies to further improve the reliability of EEG preprocessing.

Fourth, the proposed framework integrates only EEG signals and psychological features, while other physiological modalities, such as heart rate, electrodermal activity, and respiration signals, have not been incorporated. In addition, more sophisticated cross-modal interaction mechanisms remain worthy of further investigation. Future work will integrate richer multimodal physiological signals and introduce external reference criteria, including expert clinical assessments, standardized psychological diagnostic scales, and longitudinal follow-up data, to independently validate the proposed multimodal risk representation framework and further improve its reliability, interpretability, and practical applicability.

## 7 Conclusion

To address the limitations of conventional mental health risk assessment that relies heavily on single questionnaires with strong subjectivity and limited discriminative power, this study proposes a psychology-guided cross-modal attention and gated fusion network (PAG-Net). By integrating EEG physiological signals with psychological traits, the model performs cross-subject three-level risk evaluation (low, medium, and high). The framework takes 14-channel EEG as the primary neurophysiological source, employs a Graph Attention Network (GAT) to characterize topological dependencies among brain regions, and utilizes a Bi-LSTM to capture temporal evolution patterns. Under the guidance of the cross-modal attention mechanism, psychological traits steer the selection of informative EEG segments, while the gated fusion module completes adaptive complementarity between the two modalities.

Comprehensive experiments on the AMIGOS dataset demonstrate that dual-modal modeling consistently surpasses single-modal strategies. Compared with the psychology-only baseline, the EEG-only branch increases accuracy by 5.07%, and simple feature concatenation further raises it to 70.33%. In contrast, the complete PAG-Net achieves an accuracy of 74.91%, yielding the best results across accuracy, weighted F1, and related criteria, while effectively alleviating the misidentification problem in the medium-risk category. Ablation studies further confirm the rationality and cooperation of the architectural components: GAT-based spatial encoding forms the foundation of EEG representation, cross-modal attention improves inter-modal alignment and information screening, and the gated fusion layer regulates contribution balance to suppress interference from redundant cues.

Moreover, quantitative analysis of attention distributions uncovers intrinsic associations between EEG channels and mental health risk. Prefrontal and central electrodes receive consistently higher attention during prediction. Distinct contribution patterns are observed across risk groups: high-risk subjects are mainly driven by prefrontal channels, medium-risk subjects emphasize central-region activity, whereas low-risk subjects show a more homogeneous channel distribution. These findings not only enhance the interpretability of PAG-Net but also offer objective neurophysiological references for hierarchical and personalized psychological intervention design.

## Data Availability

The original contributions presented in the study are included in the article/supplementary material. Further inquiries can be directed to the corresponding author.
